# Altered Gene Expression Associated with microRNA Binding Site Polymorphisms

**DOI:** 10.1371/journal.pone.0141351

**Published:** 2015-10-23

**Authors:** Urmo Võsa, Tõnu Esko, Silva Kasela, Tarmo Annilo

**Affiliations:** 1 Estonian Genome Center, University of Tartu, Riia 23b, 51010 Tartu, Estonia; 2 Division of Endocrinology, Children's Hospital, Boston, MA, United States of America; 3 Department of Genetics, Harvard Medical School, Boston, MA, United States of America; 4 Broad Institute, Cambridge, MA, United States of America; Roswell Park Cancer Institute, UNITED STATES

## Abstract

Allele-specific gene expression associated with genetic variation in regulatory regions can play an important role in the development of complex traits. We hypothesized that polymorphisms in microRNA (miRNA) response elements (MRE-SNPs) that either disrupt a miRNA binding site or create a new miRNA binding site can affect the allele-specific expression of target genes. By integrating public expression quantitative trait locus (eQTL) data, miRNA binding site predictions, small RNA sequencing, and Argonaute crosslinking immunoprecipitation (AGO-CLIP) datasets, we identified genetic variants that can affect gene expression by modulating miRNA binding efficiency. We also identified MRE-SNPs located in regions associated with complex traits, indicating possible causative mechanisms associated with these loci. The results of this study expand the current understanding of gene expression regulation and help to interpret the mechanisms underlying eQTL effects.

## Introduction

Recent genome-wide association studies (GWAS) have identified susceptibility loci for a large number of complex diseases and traits. However, the functional interpretation of potential disease-mediating loci presents a major challenge, since only a small fraction of the associated loci has a direct effect on protein-coding regions [1]. The vast majority of GWAS-identified loci are likely to harbor regulatory variants that affect phenotypes by modulating gene expression. Mapping studies of genetic variants associated with individual differences in gene expression levels (expression QTLs or eQTLs) have revealed that the expression of most genes is influenced by multiple loci [2,3] and that signals identified by GWAS are enriched for eQTLs [4].

Gene expression regulation is a complex process, which includes genetic, epigenetic, environmental, and stochastic components, and the mechanisms underlying gene expression variation are far from understood. MicroRNAs (miRNA) are small endogenous noncoding RNAs that modulate gene expression at the post-transcriptional level. They bind to specific sequence motifs called miRNA response elements (MREs) in the 3′ untranslated region (3' UTR) of mRNAs, repressing the activity of their targets by affecting mRNA stability and/or protein translation. Recent studies have indicated that these two mechanisms are tightly coupled and that mRNA destabilization can account for more than 80% of the reduction in protein output [5–7]. Therefore, changes in mRNA expression levels can be used to directly estimate the impact of miRNAs on the cellular gene expression program [7–9].

The rapidly increasing number of known human miRNAs and trait-associated SNPs provides an opportunity to systematically investigate the potential impact of common genetic variants on regulatory interactions between miRNAs and their target mRNAs. Polymorphisms in miRNA binding sites are implicated in disease and non-pathological phenotypes, including cancer susceptibility [10], drug resistance [11], Tourette's syndrome [12], and muscle growth [13]. There is also a significant overrepresentation of GWAS-identified SNPs in the 3′ UTRs of coding genes [14], suggesting that regulatory variation within untranslated regions may play an important role in complex trait development.

Since miRNAs are important for maintaining tissue-specific transcription profiles, many genes have evolved under selective pressure to avoid target sites for simultaneously expressed miRNAs [15,16]. Despite strong selection against SNPs that either destroy conserved MREs or create new MREs in genes that avoid miRNA regulation [17,18], the human genome still contains thousands of variants that can alter miRNA binding.

Variation in gene expression levels is an intermediate step between genetic variation and complex traits. Allele-specific deregulation of gene expression due to the regulatory polymorphisms can play an important role in the development of complex disorders [19,20]. Therefore, elucidating the mechanisms by which genetic variation in regulatory regions affects gene expression remains an important question.

Our aim was to examine the impact of genome-wide MRE variation on gene expression levels and to determine whether we could identify functionally relevant genetic variants using such approach. We hypothesized that polymorphisms that either disrupt an existing miRNA binding site or create a new miRNA binding site can affect target gene expression, leading to allele-specific expression modulation. Information gained from our study could provide new insights into the functional mechanisms underlying GWAS signals and lead to an enhanced understanding of gene expression regulation in general.

## Materials and Methods

### eQTL and mRNA expression datasets

*Cis*-acting eQTLs (defined as SNPs associated with gene expression located within a 250 kb distance of the associated probe) were acquired from BloodeQTLBrowser (http://genenetwork.nl/bloodeqtlbrowser/). This browser accompanies a recent publication by Westra *et al*. [21] where eQTL meta-analysis was carried out in the peripheral blood samples of seven cohorts (n = 5,311 individuals). Data were filtered using FDR correction at a 0.05 level and 664,097 *cis*-eQTLs (unique SNP-probe pairs) were included in the current study.

A gene expression dataset (n = 914) from the Estonian Genome Center, University of Tartu (EGCUT), which was one of the cohorts from the meta-analysis, was used for visualization of the *cis*-eQTL trends. Gene expression data was obtained using Illumina HT12v3 expression arrays. Data preprocessing with the Bioconductor “lumi” package involved background-correction (method “subtract”), variance-stabilizing transformation, and quantile normalization [22].

Genotype data was obtained using Illumina Human370CNV genotyping arrays. The imputation of genotype data was carried out using SHAPEIT v1 [23] for phasing, IMPUTE v2.2.2 [24] for imputation, and 1000 Genome Phase I integrated variant set, March 2012 release reference panel.

For replication in other tissue types, GTEx v4 Data Portal was used (http://www.gtexportal.org/, [25]). *Cis*-eQTLs were calculated for top results in forty different tissues with uncorrected p-value 0.05 as a threshold for replication.

### Data preprocessing

**Fig 1:**
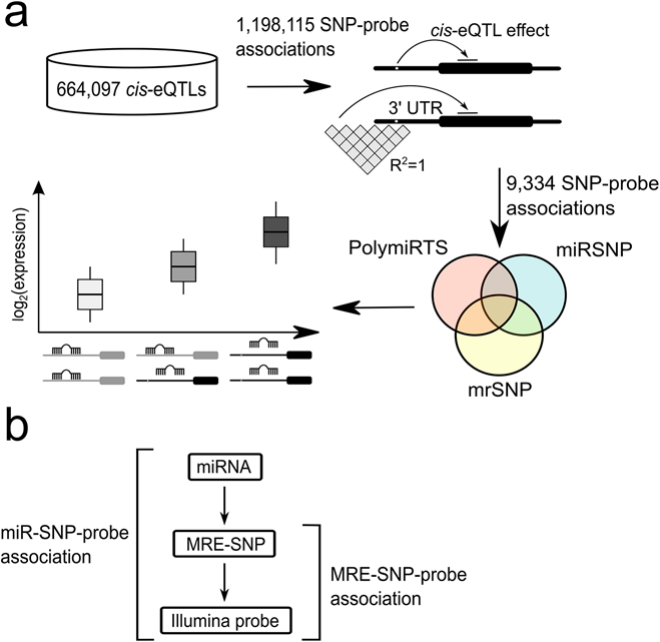
Analysis strategy for the identification of miRNA-mediated *cis*-eQTL effects. (a) *Cis*-eQTL SNPs and their perfect proxies (R^2^ = 1, 1000G CEU population) were mapped to the 3' UTRs of *cis*-affected ENSEMBL transcripts. Next, the SNPs that were located within the *in silico* miRNA binding sites were identified from public databases PolymiRTS, miRSNP, and webtool mrSNP, each utilizing a different *in silico* target prediction algorithm. The direction of allelic trends was assessed for concordance with the logic of miRNA-mediated regulation. (b) Associations between Illumina expression probes, MRE-SNPs, and miRNAs. An MRE-SNP-probe association consists of an MRE-SNP and an Illumina probe, detecting the corresponding *cis*-affected gene. An miR-SNP-probe association adds the miRNA, for which the predicted MRE is either disrupted or created by the MRE-SNP.

To identify all SNPs that are in linkage disequilibrium (LD) with *cis*-eQTL SNPs, we identified proxies with absolute LD (R^2^ = 1, 1000G pilot 1 reference panel for CEU population) and their minor allele frequencies using the SNAP v2.2 webtool [26]. SNPs not present in the reference panel were removed from subsequent analyses. The ENSEMBL v75 BioMart database was used for mapping of the *cis*-eQTL Illumina probe IDs to ENSEMBL transcripts, HUGO gene IDs, and for acquiring the corresponding 3' UTR coordinates. All genomic coordinates were standardized to hg19 (Human Genome version 19) using the liftOver tool (Bioconductor rtracklayer package). *Cis*-eQTL SNPs and their perfect proxies (R^2^ = 1) within the 3' UTRs of detected transcripts were used in subsequent analyses. An outline of the analysis is shown in Fig 1, and detailed steps are presented in S1 Fig.

### Small RNA and miRNA datasets

Small RNA sequencing datasets utilizing whole blood, peripheral blood mononuclear cells (PBMCs), or leukocytes from healthy individuals were identified by searching the Gene Expression Omnibus database (http://www.ncbi.nlm.nih.gov/geo/). We identified 11 suitable datasets from five independent studies (S1 Table). Eight of the datasets were profiled using the Illumina technology (SRR1054216, SRR1054217, SRR350239, SRR350240, SRR350241, SRR039190, SRR039191, and SRR493380) and three were profiled using the SOLiD platform (SRR580544, SRR580545, and SRR580546). SRA files were downloaded and function *fastq-dump* from the SRA Toolkit v2.3.4 [27] was used to convert them to FASTQ files. sRNABench v0.9 web version [28] was used for quality check, preprocessing, normalization, and alignment of the reads to miRBase v20 with default settings. The consensus blood-expression profile of 123 miRNAs was defined as those miRNAs identified by at least 10 reads in at least six datasets out of 11 (S2 Fig).

A “high confidence” set of precursor miRNAs was downloaded from the miRBase v20 ftp site and used for constructing the set of corresponding 502 “high confidence” mature miRNAs.

### Evaluation of the effect of MRE polymorphisms on miRNA binding

The functional impact of genetic polymorphisms on miRNA binding was obtained from three databases: PolymiRTS v3.0 (http://compbio.uthsc.edu/miRSNP/), miRSNP (http://cmbi.bjmu.edu.cn/mirsnp), and mrSNP (http://mrsnp.osu.edu/).

PolymiRTS v3.0 (timestamp Aug 27, 2013 [29]) integrates miRBase v20, dbSNP v137 and TargetScan algorithm for miRNA binding-site prediction. TargetScan [30] assumes complementarity between the target and the major determinant of miRNA effectiveness- at least 7 nt long canonical *seed* region (either 7mer-A1, 7mer-m8 or 8mer).

miRSNP (timestamp Dec 11, 2012 [31]) implements miRBase v18., dbSNP v135 and miRanda algorithm with stringent -7 nt pairing for MRE prediction. This algorithm [32] uses scoring of alignment quality and free energy for MRE identification, assuming also strict complementarity between nucleotides 2 to 8 in this setting.

mrSNP [33] is a web-based tool designed for assessing SNP effects on miRNA binding that uses a target prediction strategy based on DIANA-microT [34]. More specifically, DIANA-microT algorithm identifies MREs with perfect complementary in 7–9 nt long *seed* regions and additionally uses free energy filter for identifying binding sites with shorter 6 nt *seed* regions or those consisting G:U wobble pairs.

Default settings without conservation filters were used for all of the mentioned tools to search for SNPs that disrupt existing MREs or create new MREs.

The initial set of MRE-SNPs consisted of all of the SNPs reported in any of the three databases. The filtered set of MRE-SNPs was defined as: 1) MRE-SNPs and corresponding targeting miRNAs that existed in all three databases, 2) only miRNAs present in the consensus blood-expressed miRNA profile, and 3) the miRBase “high confidence” set (S1 Fig).

Four computational measures were used to assess the effectiveness of miRNA binding and for prioritizing putative MREs: i) Context+ score [35], which is used with the TargetScan algorithm to combine six features of each MRE and where a more negative score indicates a stronger MRE. Differences in context+ scores between wild-type and MRE-SNP-derived alleles were downloaded from the polymiRTS v3.0 database. ii) miRSVR score [36], which used the miRanda algorithm to combine seven features of each MRE and where a more negative score indicates a stronger MRE. iii) miRanda alignment score [32], which is calculated for miRNA-mRNA alignment by the miRanda algorithm and where a higher score indicates a stronger MRE. iv) Free energy, which is calculated by the miRanda algorithm to show the stability of an miRNA-mRNA duplex and where a more negative value indicates a stronger MRE. miRSVR scores, miRanda alignment scores, and free binding energy values were downloaded from the miRSNP database.

CLIP-based miRNA-target interactions (verified by high-throughput sequencing of immunoprecipitated RNAs after cross-linking) were retrieved from starBase v2.0 [37] by downloading 36 AGO1, AGO2, AGO3, and AGO4 PAR-CLIP, HITS-CLIP, and CLASH datasets (S2 Table) in BED format. Regions covered by at least one read in at least one dataset were considered AGO-CLIP-supported MREs.

Experimentally validated miRNA target sites were downloaded from miRTarBase v4.5 (http://mirtarbase.mbc.nctu.edu.tw/ [38]), TarBase v6.0 (http://diana.cslab.ece.ntua.gr/tarbase/ [39]), and miRecords v4 (http://miRecords.umn.edu/miRecords [40]), using multiMiR v1.0.1 R package [41].

The evolutionary conservation information was downloaded from polymiRTS v3.0, utilizing 46-way Multiz alignment. We classified the MRE as conserved if it was present in human and in at least two additional vertebrates.

Strand effect was taken into account by swapping allelic variants for the SNPs residing in the genes transcribed from the negative strand. Subsequently, the concordance with the expected mechanism of miRNA regulation was evaluated, using allelic direction from the meta-analysis by Westra *et al*. [21] (represented as overall Z-score). The miR-SNP-probe combinations that follow the expected miRNA-mediated regulation mechanism (i.e., the allele that destroys the miRNA binding site is associated with elevated expression of the probe, and vice versa), are referred to as "concordant-type" (C-type). And, conversely, the miR-SNP-probe combinations that do not follow the expected type of change in expression are referred to as "unconcordant-type" (U-type).

### Statistical analyses

eQTL effect sizes (overall Z-score from the meta-analysis of Westra *et al*. [21]) were averaged between correlated SNPs from the LD block (R^2^ = 1) to construct average Z-scores for each MRE-SNP-probe association. To assess the overall effect of the MRE-SNP on miRNA binding, we calculated the mean for each of the computational measures of miRNA binding site efficiency (TargetScan context+ score change, miRSVR score, miRanda binding score, and free binding energy) for each MRE-SNP-probe association.

Differences in *cis*-eQTL effect sizes and binding site efficiencies (represented as context+ score change, miRSVR score, miRanda score, and free binding energy) were compared between MRE-SNP-probe associations supporting the expected mechanism of miRNA-mediated regulation in the case of all targeting miRNAs (exclusively C-type associations), and not supporting miRNA-mediated regulation for any targeting miRNAs (exclusively U-type associations), using Wilcoxon-Mann-Whitney U-test. The Spearman correlation test was used for testing the correlation between eQTL effect sizes and mean measures of miRNA binding site efficiencies.

The Chi-square test was used to test the associations between MRE-SNP-probe concordance with eQTL effect direction (either exclusively C-type or exclusively U-type) and MRE-SNP type (exclusively MRE-disrupting or exclusively MRE-creating), MRE conservation (conserved or unconserved MRE) or AGO-CLIP data (supported or not supported). R v3.1.1 statistics software was used for analyses.

### Gene function prediction

The GeneNetwork database (http://genenetwork.nl/genenetwork/, [42]), which contains co-regulation information from ~80,000 gene expression arrays, was used for predicting the function of the top 30 filtered miR-SNP-probe associations with the strongest MREs. Functional predictions were carried out using Gene Ontology Biological Processes, KEGG, and Reactome pathways (p < 0.05 after Bonferroni correction).

### Overlap with GWAS Catalog

Trait-associated SNPs were retrieved from the NHGRI Catalog of Published Genome-Wide Association Studies (http://www.genome.gov/gwastudies) on October 6th, 2014. SNPs and their respective proxies (SNAP v2.2 webtool; R^2^ = 1, 1000G pilot 1, CEU) were searched for overlap with MRE-SNPs.

## Results

### Identification of 3' UTR-SNPs in the *cis*-eQTLs loci

Our goal was to systematically investigate the role of polymorphisms in miRNA binding motifs with regard to genome-wide gene expression regulation. In GWAS, genomic regions are identified or tagged by a very small fraction of the SNPs actually present in the genome. In animals, most of the functional miRNA binding sites are located in the 3’ UTRs of the target genes. Therefore, as a first step, we mapped the *cis*-eQTL SNPs, identified by recent comprehensive meta-analysis [21], and their proxies (R^2^ = 1) to 3' UTRs of *cis*-regulated genes.

After data filtering and processing, we identified 9,334 such 3' UTR-located *cis*-eQTL effects out of 1,198,115 total *cis*-eQTL SNPs (Fig 1 and S1 Fig). This dataset defines 61,109 SNP-transcript pairs that had either a *cis*-eQTL SNP or its proxy in the 3' UTR. Approximately 13% (7,891 out of 61,109) of these SNP-transcript pairs were detected by more than one Illumina probe. Surprisingly, for ~15% (1,186 out of 7,891) of these multi-probe SNP-transcript pairs, the *cis*-effects of probes detecting the same transcript showed discordant *cis*-eQTL effect directions. This may suggest possible alternative splicing or polyadenylation in at least some of those genes [43]. Since the omission of these transcripts (which accounted for less than 2% of the total SNP-transcript pairs) would have biased the dataset, they were not excluded from further analyses.

We also observed discordant *cis*-eQTL effect directions between individual SNPs in two LD blocks, which were removed from subsequent analyses.

### MRE-SNPs can create and disrupt miRNA binding sites

To identify SNPs with the potential to disrupt existing MREs or create new MREs (referred to as MRE-SNPs), we used three databases utilizing different miRNA target prediction algorithms (PolymiRTS, miRSNP, and mrSNP). We identified 27,126 SNP-probe combinations involving a *cis*-acting eQTL SNP or its proxy in the predicted MRE of a *cis*-regulated gene, accounting for approximately 4% of all *cis*-eQTLs identified by Westra *et al*. [21].

These combinations involved 5,992 *cis*-eQTL SNPs and proxies (R^2^ = 1) in the putative MREs of 2,545 genes, which corresponds to 72% of all 8,318 SNPs and proxies mapped to 3' UTRs of affected genes (S1 Fig, S1 Dataset). The expression of those genes was detected by 2,882 unique Illumina probes, which made up ~35% of 8,201 *cis*-eQTL-associated probes identified by Westra *et al*. [21].

The binding of 2,573 miRNAs out of 2,578 annotated in miRBase v20 was predicted to be influenced by at least one of the SNPs. Altogether, 31,725 putative miR-SNP-probe associations were identified (panel b in S1 Fig).

The average number of MREs affected per SNP was 4.72. The rs2066934 SNP in the 3' UTR of *CSF1R* was predicted to affect the binding of the largest number of miRNAs, with a total of 40 associated miRNAs. The average number of MRE-SNPs per miRNA was 11.15. The miR-548c-3p miRNA was predicted to be affected by the largest number of MRE-SNPs, with a total of 37 associated MRE-SNPs. The minor allele disrupted the predicted MRE in 14,153 cases, and created a new MRE in 14,250 cases. Among the MRE-SNPs, 1,137 were exclusively MRE-disrupting, 1,191 were exclusively MRE-creating, and 3,664 were both MRE-disrupting and MRE-creating.

### Analysis of MRE-SNPs and concordance with miRNA-mediated regulation

#### Unfiltered analysis

Hypothesizing that a functional miRNA-mRNA interaction results in the down-regulation of the target transcript, we assessed the possibility of an miRNA-associated mechanism of *cis*-eQTL by using the allelic direction from the meta-analysis by Westra *et al*. [21]. We refer to combinations where the expression change follows the miRNA-mediated mechanism as "concordant" or "C-type" and to combinations with the opposite effect as "unconcordant" or "U-type". We observed that the allelic direction was concordant with our hypothesis in the case of 15,682 (49.4%) miR-SNP-probe associations and unconcordant in the case of 16,043 (50.6%) miR-SNP-probe associations.

Out of 6,727 MRE-SNP-probe associations, 19.5% (1,312) were exclusively concordant (miRNA-mediated regulation supported by all targeting miRNAs), 19.6% (1,321) were exclusively unconcordant, and 61% (4,094) were involved in both concordant and unconcordant miRNA targeting. The average effect size (expressed as the Z-score in [21]) was not significantly different between exclusively concordant SNP-probe associations and exclusively unconcordant SNP-probe associations (Wilcoxon-Mann-Whitney U-test, p > 0.05, panel a in S3 Fig), and we did not observe any association in the case of exclusively MRE-breaking or exclusively MRE-creating SNPs separately (Wilcoxon-Mann-Whitney U-test, p > 0.05, panels b and c in S3 Fig). Concordant SNPs were not overrepresented among either exclusively MRE-breaking SNPs or exclusively MRE-creating SNPs (Chi-square test, p > 0.05).

#### Filtering of the MRE-SNP dataset

To apply more stringent criteria to our analyses, we filtered the MRE-SNP dataset based on three conditions: i) conservative use of prediction methods including the intersection of three databases, ii) use of miRNAs expressed in blood, based on publicly available small RNA sequencing datasets, since the original eQTL mapping was performed using peripheral blood samples [21], and iii) the quality of miRNA annotations, as provided by the "high confidence" miRNA dataset available at miRBase v20. As one of the MRE-SNP databases, polymiRTS 3.0, utilizes TargetScan algorithm for target prediction, the resulting set of MRE-SNPs influences the miRNA-mRNA pairing only in the most crucial part of the duplex- canonical *seed* region (either 8mer, 7mer-m8 or 7mer-A1).

**Fig 2:**
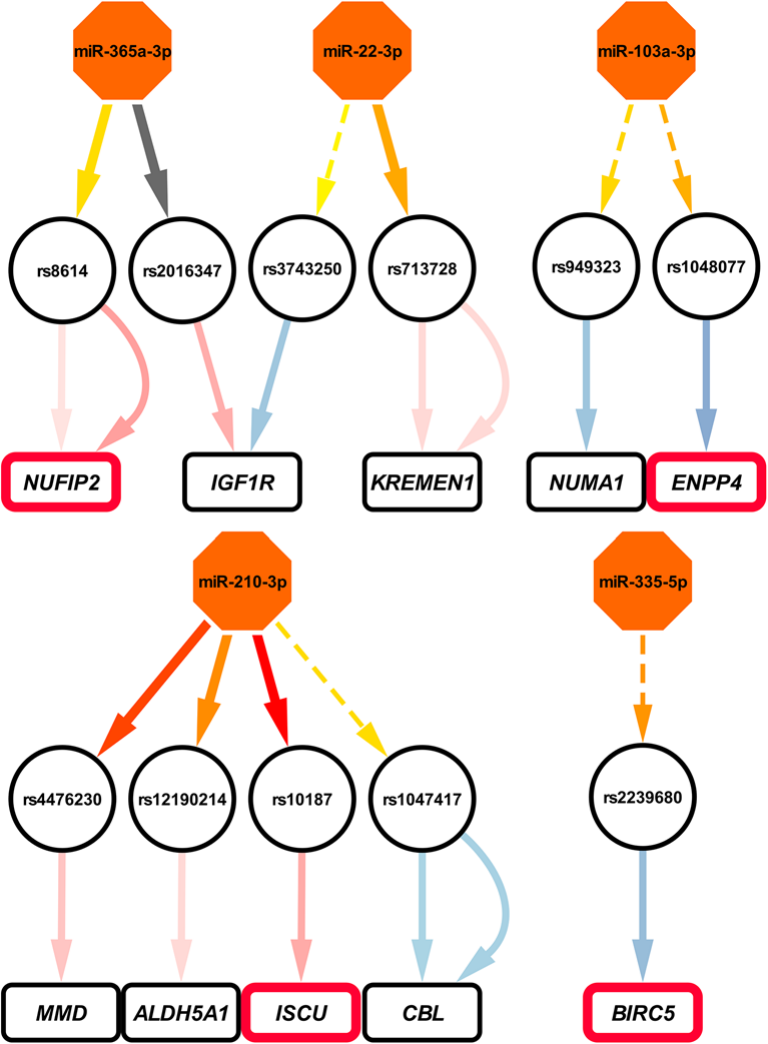
Graphs depicting miRNA-SNP-mRNA associations. Shown is an example subset of filtered associations supported by AGO-CLIP data (see S4 Fig for full data). The color of miRNA-SNP links shows miRNA targeting probability, assessed by TargetScan context+ score change (grey, context+ score change > 0; the gradient from yellow to red shows the magnitude of context+ score change) and the line type indicates concordance with the mechanism of miRNA-mediated regulation: a continuous line is concordant (C-type), and a dashed line is unconcordant (U-type). In the case of SNP-gene links, the concordance type is indicated by the color: a red line is C-type, a blue line is U-type, and the intensity of the color indicates the *cis*-eQTL effect size (overall Z-score) from the Westra *et al*. meta-analysis [21], averaged over all detected SNPs in the LD block (R^2^ = 1). Multiple interactions between one SNP and one gene show several Illumina probes detecting the same transcript. Outlined genes are validated targets of a targeting miRNA, as reported by miRTarBase, TarBase, or miRecords.

Applying these criteria, we identified a filtered set of 323 miR-SNP-probe associations consisting of 217 MRE-SNPs, 57 miRNAs, and 241 probes corresponding to 206 genes (Table 1, S3 Table, Fig 2 and S4 Fig). Of them, the minor allele was disrupting 163 MREs and creating 160 MREs.

**Table 1:** Top 30 filtered SNP-miR-probe associations, sorted based on their context+ scores.

MRE-SNP	Effect^a^	Alleles	MAF^b^	No. of eQTL proxies^c^	Illumina probe	MeanZ-Score^d^	Gene	miRNA	Context+ score	miRSVR	miRanda score	Type^e^
**rs10187**	D	C/T	0.06	1	ILMN_2409062	13.39	*ISCU*	miR-210-3p	-0.55	-1.10	154	C
rs11932	D	C/T	0.17	16	ILMN_1676005	5.69	*KPNA1*	miR-210-3p	-0.47	-0.22	152	U
rs7676	C	T/C	0.19	6	ILMN_1682812	7.17	*C21orf33*	miR-423-5p	-0.45	-0.72	145	C
					ILMN_1737588	7.01						C
					ILMN_2386205	10.11						C
rs4476230	C	T/G	0.47	1	ILMN_1733937	8.92	*MMD*	miR-210-3p	-0.42	n.a.	147	C
rs2781667	D	C/T	0.28	6	ILMN_1674506	4.79	*MED23*	miR-361-3p	-0.35	-0.92	152	U
**rs4245739**	C	A/C	0.32	1	ILMN_1746020	18.27	*MDM4*	miR-191-5p	-0.32	-0.61	155	C
rs2255015	C	G/A	0.48	1	ILMN_1688464	23.48	*MAP6D1*	miR-148a-3p	-0.32	n.a.	144	C
								miR-148b-3p	-0.32	n.a.	150	C
rs444271	C	G/C	0.12	1	ILMN_2345142	6.56	*SULF2*	miR-140-3p	-0.31	-0.23	145	U
rs1136808	D	G/C	0.41	16	ILMN_1743032	26.67	*CTSS*	miR-423-5p	-0.31	-0.13	146	C
rs10039796	C	C/G	0.48	1	ILMN_1658962	4.29	*LCP2*	miR-365a-3p	-0.29	-0.84	162	U
**rs17765088**	D	C/G	0.13	1	ILMN_2337386	4.79	*CCR9*	miR-335-5p	-0.29	-0.33	153	U
rs6728	D	C/G	0.26	4	ILMN_1694084	12.54	*CYTH1*	miR-10a-5p	-0.29	-0.97	153	U
					ILMN_2403852	5.66						U
**rs3828609**	D	T/C	0.15	3	ILMN_1686623	17.91	*CSF1R*	miR-155-5p	-0.28	-1.01	148	U
rs12190214	D	C/A	0.13	1	ILMN_2372398	5.65	*ALDH5A1*	miR-210-3p	-0.28	-0.80	140	C
rs11730	D	A/G	0.47	7	ILMN_1739541	25.69	*NMI*	miR-148a-3p	-0.28	-1.18	154	C
								miR-148b-3p	-0.28	-1.18	146	C
rs1135314	C	A/G	0.43	1	ILMN_1712532	26.69	*CARD9*	miR-148a-3p	-0.27	n.a.	145	U
								miR-148b-3p	-0.27	n.a.	146	U
rs1057560	C	A/G	0.50	1	ILMN_1732489	14.49	*SLC10A7*	miR-25-3p	-0.26	-0.31	140	C
rs73215937	C	T/C	0.17	21	ILMN_1758323	20.64	*ACPP*	miR-486-3p	-0.26	n.a.	150	C
rs13936	D	G/A	0.07	1	ILMN_1789909	4.07	*TBC1D9B*	miR-296-5p	-0.26	-0.12	140	C
rs2287067	C	T/C	0.17	1	ILMN_1699275	4.13	*VWDE*	miR-590-5p	-0.26	n.a.	155	C
**rs2239680**	D	T/C	0.26	3	ILMN_2349459	8.62	*BIRC5*	miR-335-5p	-0.26	-0.63	157	U
rs10438593	C	C/T	0.12	1	ILMN_1699607	8.77	*MMP25*	miR-181d-5p	-0.25	n.a.	165	U
					ILMN_1717207	6.40						U
rs1057560	C	A/G	0.50	1	ILMN_1732489	14.49	*SLC10A7*	miR-92a-3p	-0.25	-0.31	140	C

SNPs shown in bold are in experimentally validated MREs.

^a^ SNP effect on miRNA binding: D disrupts an MRE and C creates an MRE.

^b^ MAF is based on 1000G pilot 1 CEU population.

^c^ Number of *cis*-eQTL SNPs from the LD block (R^2^ = 1, 1000G pilot 1, CEU)

^d^ Z-scores from a recent meta-analysis [21] are averaged for LD block (R^2^ = 1); FDR < 0.05 for all associations.

^e^ Association type: C-concordant; U-unconcordant.

Out of the 254 MRE-SNP-probe pairs, 130 (51%) were concordant for all of the targeting miRNAs, and 117 (46%) were unconcordant for all of the targeting miRNAs. Additionally, for seven MRE-SNP-probe pairs (3%), the minor allele replaced one set of MREs with another such that they were only concordant with a subset of targeting miRNAs. Four *cis*-affected genes had opposite *cis*-eQTL effects, depending on the Illumina probe used to detect the transcripts of the same gene. We did not observe any difference in effect sizes between exclusively concordant and exclusively unconcordant sets of MRE-SNP-probe associations (Wilcoxon-Mann-Whitney U-test, p > 0.05, S5 Fig).

Out of 323 filtered miR-SNP-probe associations, 194 influenced conserved MRE. However, there was no enrichment of C-type associations among conserved binding sites compared to the non-conserved binding sites (Chi-square test, p > 0.05).

Next, we analyzed whether the C-type *cis*-eQTLs are associated with functionally more effective MREs by testing the impact of parameters characterizing the relevance of the miRNA regulatory potential. To assess the effectiveness of each MRE-SNP for the binding of all targeting miRNAs, we calculated average effectiveness scores for each MRE-SNP-probe pair (as described in the Materials and Methods). We did not find any association between miRNA regulation concordance and miRNA binding effectiveness, as determined by the mean context+ score change, mean miRSVR score, and mean free binding energy (Wilcoxon-Mann-Whitney U-test, p > 0.05, S6 Fig). However, there was a marginally lower mean miRanda score in the C-type group (Wilcoxon-Mann-Whitney U-test, p = 0.049), although we would expect higher scores among MREs with stronger biological effects. There was no correlation between the magnitude of the effect and miRNA binding efficiency, aside from a significant negative association between mean miRanda score and effect size exclusively in the U-type MRE-SNP-probe set (Spearman correlation test, p = 0.024, panel c in S7 Fig).

miRNAs with the largest number of SNPs in the MREs included miR-361-3p and miR-340-5p, with 14 predicted MRE-SNPs (panel c in S4 Fig). MRE-SNPs affecting the binding of the largest number of miRNAs included rs7520333 in *RIMS3* (predicted MRE for 7 miRNAs), rs1266479 in *MAN2A2* (predicted MRE for 6 miRNAs), and rs2521795 in *GDE1* (predicted MRE for 6 miRNAs) (panel a in S4 Fig). Among them, rs7520333 is targeted primarily by the highly expressed let-7 family miRNAs, but the direction of the *cis*-eQTL effect is U-type. rs2521795 and rs1266479 are both potentially targeted by members of the miR-17-92 family and several other miRNAs (panel a in S4 Fig). These two SNPs are also located in the experimentally identified AGO-binding sites (according to starBase database), supporting the presence of functional MREs in these locations. The functional miRNA-mediated eQTL effect between rs2521795 and *GDE1* is exclusively C-type, and is fully supported by the results of meta-analysis by Westra *et al*. [21] (FDR < 1.6 × 10^−5^, Z-score = 9.39).

To assess the biological functions of the genes harboring the strongest MRE-SNPs in a hypothesis-free manner, we utilized a co-expression network constructed from ~80,000 expression arrays [42]. Associated genes were involved in a wide variety of pathways and processes (S4 Table, S8 Fig). For example, among the genes with the strongest C-type associations, *ISCU* was associated with cellular respiration and neurological diseases, *C21orf33* was associated with metabolic pathways, and *MMD* was associated primarily with immune response.

### MRE-SNPs supported by the AGO-CLIP dataset and validated miRNA targets

To integrate additional information about the validity of miRNA-target interactions into our analysis, we first used publicly available AGO-CLIP data [37]. The AGO-CLIP technology identifies Argonaute-bound miRNAs and transcriptome-wide Argonaute binding sites based on immunoprecipitation of RNA-Argonaute complexes. We found that 1,490 MRE-SNPs (~25% of all SNPs from the unfiltered dataset) are covered by AGO-CLIP peaks identified in different cell lines. However, we observed no enrichment of C-type miR-SNP-probe associations among AGO-supported MRE-SNPs (Chi-square test, p > 0.05).

Next, we defined which MRE-SNPs affect miRNA-mRNA associations supported by the experimentally validated target databases. Using TarBase, miRTarBase, and miRecords, we found that only 51 (0.85%) MRE-SNPs from our initial unfiltered set of 5,992 MRE-SNPs affect validated targets in any of these databases. However, the fraction of validated targets was more than seven times higher in the filtered set of *cis*-eQTL associations (6.5%, or 14 out of 217 SNPs, Table 2).

**Table 2:** MRE-SNPs from the filtered set of associations that overlap with validated miRNA-target interactions.

MRE-SNP	Effect^a^	Allele	MAF^b^	Gene	MeanZ-score^c^	miRNA	Seed	Conservation^d^	AGO^e^	Context+change	miRSVR	miRandascore	Validation^f^	Ref	Type^g^
rs10187	D	C/T	0.06	*ISCU*	13.39	miR-210-3p	8mer	mammals	+	-0.55	-1.10	154	IM/EX/QP	[44]	C
													LU/QP/WE	[45]	
													CL	[46]	
rs4245739*	C	A/C	0.32	*MDM4*	18.27	miR-191-5p	7mer-m8	no	-	-0.32	-0.61	155	LU	[47]	C
rs17765088	D	C/G	0.13	*CCR9*	4.79	miR-335-5p	8mer	mammals	-	-0.29	-0.33	153	EX	[48]	U
rs3828609	D	T/C	0.15	*CSF1R*	17.91	miR-155-5p	8mer	mammals	-	-0.28	-1.01	148	EX	[49]	U
rs2239680*	D	T/C	0.26	*BIRC5*	8.62	miR-335-5p	7mer-m8	no	+	-0.26	-0.63	157	LU	[50]	U
rs1048077	D	G/A	0.34	*ENPP4*	12.00	miR-103a-3p	8mer	no	+	-0.19	n.a.	147	SE	[51]	U
rs1056801	D	T/C	0.08	*SEPT2*	3.88	miR-20a-5p	8mer	primates	+	-0.11	-1.01	149	CL	[46]	C
						miR-17-5p	8mer	primates	+	-0.10	-1.02	146	CL	[46]	C
rs8614	D	C/A	0.11	*NUFIP2*	4.01	miR-365a-3p	8mer	vertebrates	+	-0.08	-0.02	169	CL	[46]	C
					15.20										C
rs2241201	D	C/G	0.24	*MMAB*	4.40	miR-335-5p	7mer-m8	no	-	-0.08	-0.02	143	EX	[48]	U
rs2341984	C	C/T	0.25	*SLC6A6*	10.58	miR-423-5p	8mer	no	-	-0.07	-0.01	156	CL	[46]	U
rs2151511	C	G/A	0.48	*MTG2*	7.66	miR-769-5p	7mer-m8	mammals	-	-0.07	-0.01	144	CL	[46]	U
rs710100	C	G/A	0.39	*TNFAIP2*	6.68	miR-155-5p	7mer-A1	primates	-	0.04	-0.01	149	PS	[52]	U
rs17759843	C	G/A	0.09	*PPM1F*	10.31	miR-186-5p	7mer-m8	primates	-	0.08	n.a.	152	CL	[46]	U
rs4679372	D	A/G	0.29	*ZNF148*	20.39	miR-186-5p	7mer-m8	primates	-	0.13	n.a.	156	SE	[51]	C

* SNP has been experimentally shown to influence MRE.

^a^ SNP effect on miRNA binding: D disrupts an MRE and C creates an MRE.

^b^ MAF is based on 1000G pilot 1 CEU population.

^c^ Z-scores from a recent eQTL meta-analysis [21] are averaged for LD block (R^2^ = 1, 1000G pilot 1, CEU); FDR < 0.05 for all associations.

^d^ Conservation shows in which species a corresponding MRE is present (polymiRTS v3.0 database).

^e^ Supported by AGO-CLIP data.

^f^ Validation method: LU- luciferase reporter assay; IM- immunoprecipitation; EX- target expression profiling; CL- CLASH; QP- qRT-PCR; SE- sequencing; PS- pSiLAC; and WE- Western blot.

^g^ Association type: C-concordant; U-unconcordant.

Although we did not observe any enrichment of C-type MRE-SNPs among the variants overlapping with validated targets, some of the associations with the highest metrics of miRNA efficiency are supported by both *cis*-eQTL data and functional validation, and may therefore be functional. For example, the SNP breaking the MRE with the smallest context+ score of -0.568 among validated targets is rs7164, which is located in the predicted binding site of miR-744-5p. The affected gene, *DDX23*, is involved in RNA processing and splicing.

**Fig 3:**
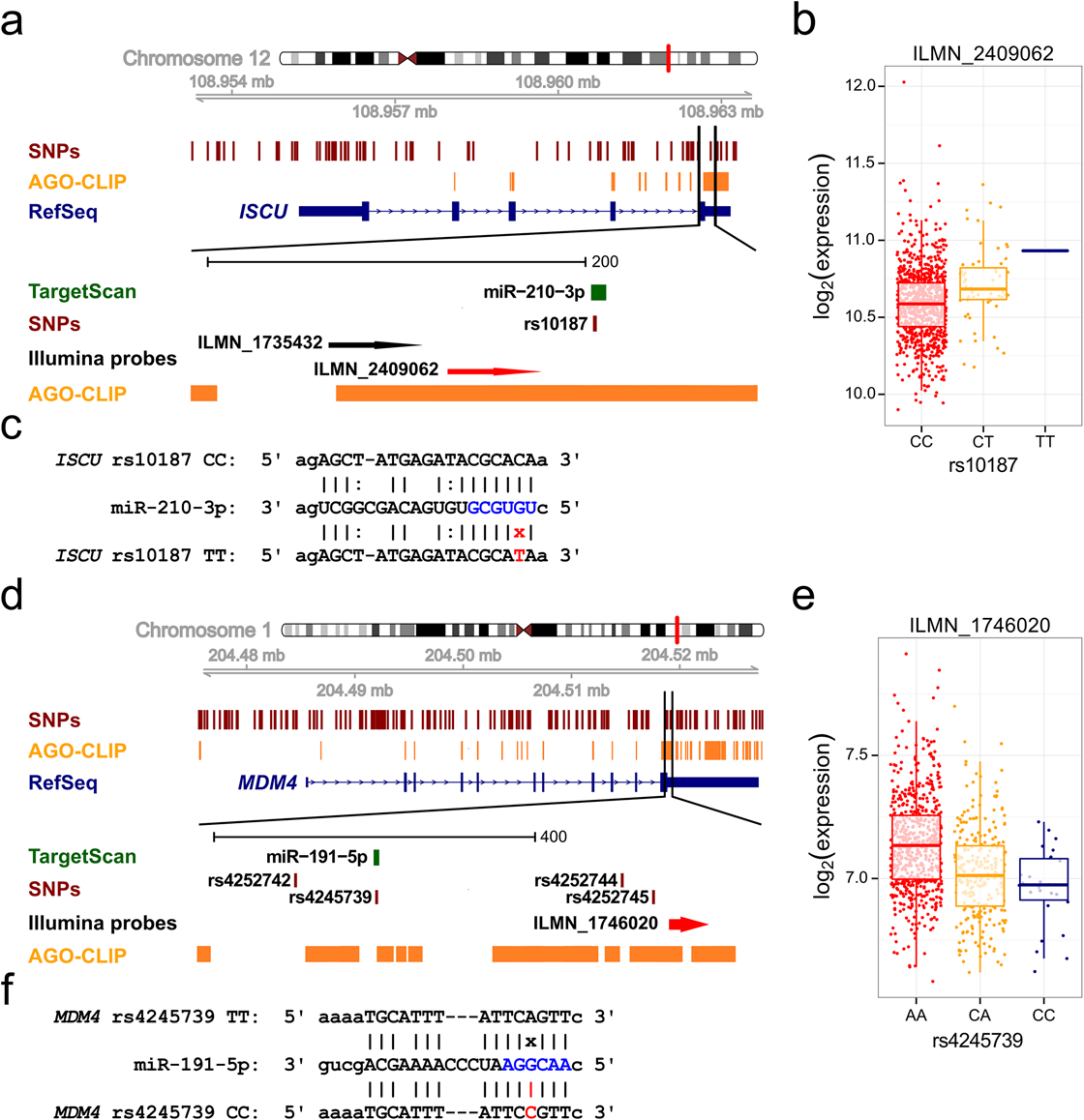
MRE-SNPs in the *ISCU* and *MDM4* genes. (a), (d) A schematic depiction of each gene locus. Tracks denote the genomic region, SNP positions, AGO binding sites from starBase, most prevalent RefSeq transcript isoform, miRNA target sites from TargetScan v6.2, and Illumina detection probes. The region with the *cis*-eQTL MRE-SNP and affected probe (marked red) is expanded. (b), (e) Allelic expression pattern in the EGCUT sample set. Log-transformed and quantile-normalized expression values are visualized as boxplots, and individual expression values are depicted as points. On the boxplots, the line indicates median, box defines 25% - 75% quartiles and whiskers extend the data to 1.5× of interquartile range. (c), (f) MRE-SNP effects on miRNA binding. Vertical lines indicate canonical pairing, colons depict G:U wobble, and "x" denotes a mismatch. The miRNA seed region is shown in blue, and the MRE-SNP minor allele and its effect on miRNA binding is shown in red.

From the filtered set, the MRE-SNP with smallest context+ score of -0.554 is rs10187, for which the minor allele disrupts the conserved 8mer binding site of miR-210-3p in the *ISCU* gene (Table 2, Fig 3A and 3C). This binding has been validated by several groups [44–46] and is located in the binding site of AGO proteins.

We also identified two MRE-SNPs that have been shown to influence miRNA binding in experimental settings. The minor allele of the C-type SNP rs4245739 creates the strong 7mer-m8 MRE (context+ score -0.318) for miR-191-5p in *MDM4* (Fig 3D and 3F). Although it is not directly covered by an AGO-CLIP peak, there is a peak located in the immediate vicinity (Fig 3D) and the effect of the SNP on miR-191-5p binding has been experimentally verified in cell lines [47].

The minor allele of U-type rs2239680 disrupts the strong 7mer-m8 MRE (context+ score -0.26) for the miR-335-5p in *BIRC5*. Although the observed *cis*-eQTL effect in blood is smaller than in case of *MDM4* and the direction does not support the logic of miRNA-mediated regulation, this MRE-SNP is supported by AGO-CLIP data and its effect on miRNA binding has been experimentally validated [50].

### miRNA-associated *cis*-eQTL replication in other tissues

To investigate whether the potential miRNA-mediated *cis*-eQTL effects identified from blood replicate in other tissues, we utilized publicly available data from GTEx Project [25]. There were several C-type associations from the top lists showing the same direction and nominally significant *cis*-eQTL effect in multiple tissues (S9 Fig). For example, *cis*-eQTL effect between rs2287067 and *VWDE* was also present in 12 out of 40 GTEx tissues. The *cis*-eQTL effect between rs4245739 and *MDM4* was also present in several tissues, including GTEx dataset for whole blood.

### Impact of complex trait-associated SNPs within MREs

Based on our hypothesis that genetic variants that modulate gene expression are likely to influence the development of phenotypic traits or disease risk, we searched for overlap between MRE-SNPs and published GWAS loci with their respective perfect proxies.

**Fig 4:**
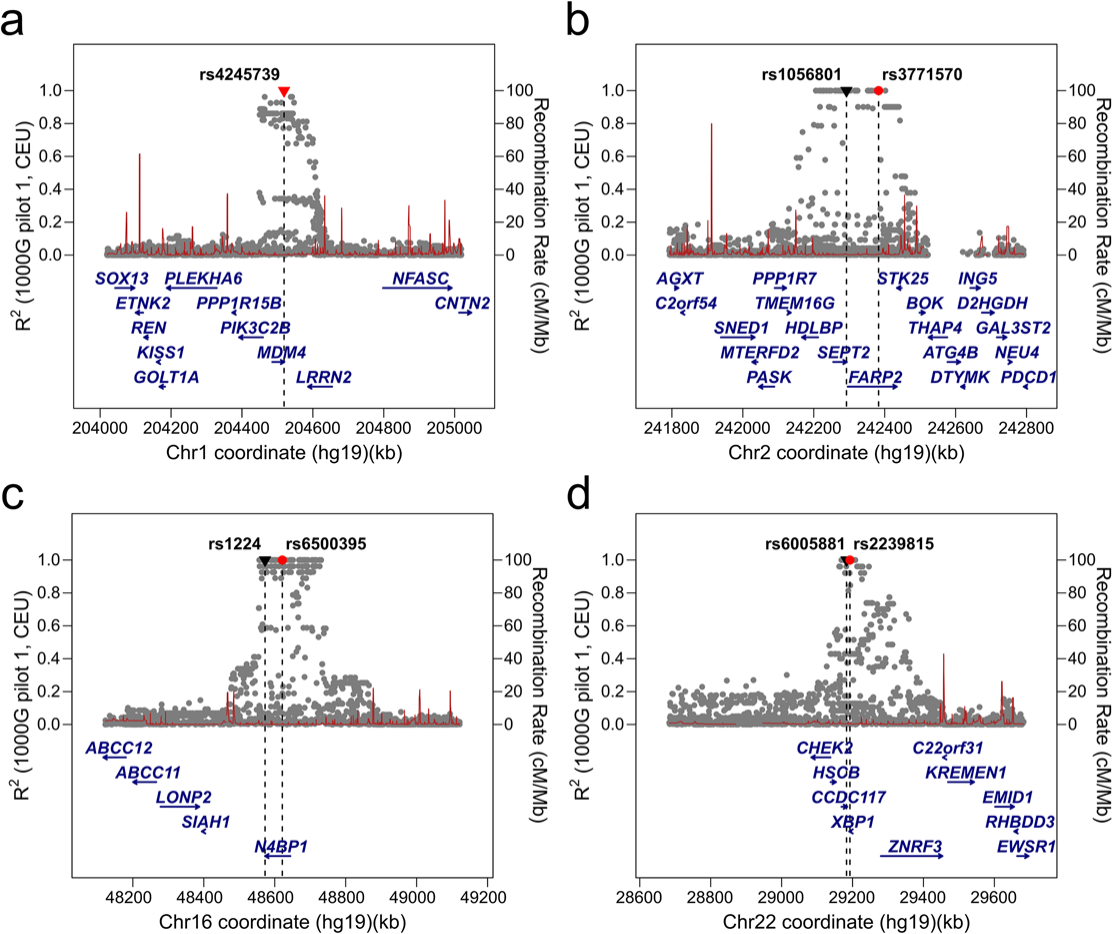
Regional LD plots for the four C-type MRE-SNPs overlapping with the GWAS Catalog.

Comparing 5,992 putative MRE-SNPs and their proxies against the catalog of published GWAS associations revealed overlap between 208 (3.5%) MRE-SNPs and 154 GWAS SNPs or their proxies. The filtered set of MRE-SNPs contains 10 (4.6%) variants, associated with 12 traits that overlap with GWAS hits (S5 Table). Four of these variants were C-type (Fig 4).

## Discussion

For most of the trait-associated loci identified by GWAS, pinpointing the causative variant has been a major challenge. Often, the associated SNPs are located outside of the coding regions, indicating that they are involved in modulating spatiotemporal gene expression, rather than protein structure. Previous work has shown a significant overrepresentation of trait-associated SNPs in 3' UTRs, highlighting the potential significance of post-transcriptional regulation [14]. Here, we provide a systematic survey of 3' UTR variants and examine their potential influence on mRNA expression levels via miRNA binding. We used the largest and most robust *cis*-eQTL dataset available, identified from the peripheral blood of 5,311 individuals from seven independent cohorts [21]. To the best of our knowledge, the present study is the first to systematically investigate the impact of MRE polymorphisms on a genome-wide scale by taking into account the direction of expression changes and by incorporating the public eQTL, miRNA expression profiling, and AGO-CLIP data into the analysis.

Although it is estimated that >10% of the total length of all 3' UTRs is covered by *in silico* predicted miRNA target sites [18], the extent to which gene expression variation is mediated by miRNA binding alterations has not been determined. SNPs located in MREs may influence mRNA expression post-transcriptionally by disrupting or creating functional miRNA binding sites, by changing the effectiveness of MREs, or by replacing the binding site of one miRNA with that of another.

Previous studies have identified a number of SNPs with the potential to influence miRNA binding. Some of the results of these studies are summarized in online databases [18,31,33,53,54], however the sensitivity and specificity of *in silico* target prediction algorithms are not perfect [55], and additional support is needed to validate an miRNA target site such that it is useful for identifying functionally relevant MRE-SNPs.

Limiting putative MRE-SNPs to the subset overlapping with Argonaute binding sites [14] may add confidence to *in silico* predictions. However, it is noteworthy that there are significant differences between individual AGO-CLIP datasets, possibly due to heterogeneity between cell types studied. Several studies have also utilized the MRE-SNPs from experimentally supported target sites [56,57] or the co-expression of the target and corresponding miRNA [56,58,59].

So far, only a handful of studies have systematically investigated the link between eQTLs and MRE-SNPs. Landmark study by Richardson *et al*. utilized miRanda algorithm for MRE-SNP identification, applying miRNA-target co-expression and SNP presence in GWAS Catalog as additional filters [60]. Out of 39 filtered associations they identified 4 which showed marginally significant eQTL trend, supporting the logic of miRNA regulation.

Gamazon *et al*. used lymphoblastoid cell lines (LCL) from 60 European (CEU) and 60 Yoruban (YRI) individuals to analyze genetic variants, mRNA, and miRNA expression data [61]. They identified a number of putative MRE-SNPs by using *in silico* target prediction algorithms, assuming a negative miRNA-target correlation and eQTL effect on the target expression. They reported that 18–25% of SNPs having *trans*-eQTL effects on miRNA expression also have associations with mRNA levels, indicating the importance of genetic variants on miRNA regulatory networks.

In another study, the authors integrated available genomic and gene expression information from 149 HapMap 3 LCL samples to identify 2,262 putative MRE-SNPs, of which 5.74% also had *cis*-effects on their target genes [62].

In a more focused study, Wei *et al*. [63] intersected *cis*-eQTLs previously identified from LCL, liver, and brain with *in silico* predicted MREs from a limited set of 409 xenobiotic-associated genes and identified 27 putative MRE-SNPs.

In the present study, we identified 5,992 putative MRE-SNPs, which we narrowed down to a prioritized set of 217 MRE-SNPs using more stringent criteria. In either case, we did not detect any significant overrepresentation of MRE-SNPs for which the *cis*-eQTL direction and effect on miRNA binding would be concordant. The effect sizes did not differ between exclusively concordant and exclusively unconcordant *cis*-eQTLs, and, likewise, we did not observe any significant association between the average effect of a given MRE-SNP and the *cis*-eQTL effect size.

Ambiguity in our results can be explained by several factors. First, most of the 3' UTRs contain binding sites for several different miRNAs, and quite often, there is more than one site for a specific miRNA. This means that the effect of disrupting or creating a single binding site may be reduced by the action of other sites. Second, the effect of a 3' UTR SNP can be manifested through different mechanisms, since both miRNA binding and mRNA stability in general are affected by several different factors. These mechanisms may include alternative polyadenylation or splicing, mRNA structural alterations, or the accessibility to RNA-induced silencing complex (RISC). Some of those mechanisms were addressed in a recent study [14], suggesting that the majority of 3' UTR SNPs influence MREs rather than splicing sites or 3' UTR folding. Third, miRNAs and their targets form complex regulatory modules that contain feedback loops to minimize the effects of genetic variation and stochastic noise [64,65]. Fourth, miRNA-mediated effects on target expression are rapid in time but modest in magnitude and may therefore escape detection. In addition, the major biological role of miRNAs seems to be to dampen the stochasticity of gene expression [66]. Therefore, the loss of an miRNA site may not result in a change in expression level, but rather an increase in expression variability.

Although we were not able to detect a statistically significant enrichment of MRE-SNPs with concordant-type regulation in our analyses, we did uncover several interesting candidates where miRNA-mediated regulation may take place. The SNP with one of the most striking effects on its predicted miRNA-binding site is rs10187, which disrupts the MRE of miR-210-3p in the *ISCU* gene. As the site is covered by AGO-CLIP reads and targeting is experimentally well validated [44–46,67], we have reason to believe that this miRNA activity may be the underlying cause of the observed *cis*-eQTL effect. Interestingly, a significant *cis*-eQTL effect is present for only one out of two Illumina probes detecting *ISCU*, demonstrating possible technical variability in expression datasets. *ISCU* codes a protein with a functional role in the assembly of mitochondrial iron-sulfur clusters. Its decreased mRNA level has been associated with myopathy with exercise intolerance [68] and decreased cancer survival [69]. However, as the minor allele of rs10187 correlates with increased expression of *ISCU*, a direct link between the MRE-SNP and these conditions is unclear. miR-210-3p is a hypoxia-inducible miRNA (hypoxamir) controlled by hypoxia inducible factors (*HIF*, for a review see [70]) and, among other functions, down-regulates mitochondrial metabolism under hypoxic conditions by directly targeting *ISCU* [69].

Two potential MRE-SNPs—rs4245739 in *MDM4* (creating an MRE for miR-191-5p) and rs2239680 in *BIRC5* (disrupting the MRE for miR-335-5p)—have been experimentally shown to affect miRNA binding in cell lines [47,50]. Interestingly, only rs4245739 has a C-type *cis*-eQTL effect in peripheral blood. The lack of concordance between the allelic trend of rs2239680 and the expected effect on miRNA-mediated regulation may be explained by several factors: miR-191-5p is one of the most highly expressed miRNAs in blood (detectable in nine out of 11 blood samples) and represents ~1% of the detected miRNome, whereas miR-335-5p is detectable in only six out of 11 blood samples and represents roughly 0.01% of the detected miRNome (S2 Fig). At the same time, miR-335-5p has a significantly larger *in silico* predicted targetome than miR-191-5p (3,046 target genes versus 568 target genes, respectively; TargetScan v6.2), suggesting that the effect of miR-335-5p on any single MRE may be diluted in this tissue.

Our study also has several limitations. The primary analysis was restricted to a single tissue type: peripheral blood. Since miRNAs are known to play a role in development and often play a very specific role in a particular tissue, it is likely that similar studies focused on other tissue types will reveal additional information about the role of genetic variation in MREs. We investigated the heterogeneity of *cis*-eQTLs by using the independent data from GTEx project. Although the sample sizes and resulting replication power are currently much smaller than in blood meta-analysis, we demonstrated that several exclusively C-type MRE-SNPs have also nominally significant *cis*-eQTL effect in other tissues than blood. Unfortunately, rs10187 was not available in GTEx datasets but concordant *cis*-eQTL effect between rs4245739 and *MDM4* was also present in several other tissues than blood, suggesting the widespread action of this miRNA-mediated mechanism in multiple tissue types (S9 Fig).

The levels of miRNA expression also vary between individuals, and it is likely that further analyses comparing SNP, gene expression, and miRNA expression data collected from the same individuals would be helpful. In addition, our analysis relies to a large extent on computationally predicted miRNA target motifs.

To overcome some of the limitations, we applied several filters to our dataset. To minimize the effect of tissue specificity of miRNA expression, we defined a blood-expressed miRNome using publicly available data. Due to the limitations of *in silico* target prediction algorithms, we also used the intersection of miRNA target predictions from three databases and the set of miRBase “high confidence” mature miRNAs. Although we integrated the information from experimentally validated targets into our analyses, it should be noted that an experimentally confirmed MRE does not necessarily provide solid proof of the functional consequence of an MRE-SNP on miRNA binding and its subsequent effect on mRNA and protein levels. Regardless of these limitations, our analysis identified four trait-associated concordant-type MRE-SNPs as a proof of concept, for which three variants were related to cancer.

One of the most interesting SNPs from the results of our filtered analysis is the aforementioned rs4245739 (A/C), which creates a functionally verified MRE for miR-191-5p in *MDM4* (Fig 3D and 3F, Fig 4A). The *MDM4*-encoded protein inhibits p53 post-translationally and is upregulated in tumors [71,72], while the minor allele of rs4245739, carried by approximately 20% of the general population, is associated with a protective effect for several cancers [47,73–75] and may serve as a potential biomarker. Most importantly, the effect of rs4245739 on miR-191-5p binding and subsequent down-regulation of *MDM4* mRNA and protein expression has been experimentally verified in ovarian cancer cell lines [47], serving as an example of a functional MRE-SNP identified independent of our systematic genome-wide approach.

The second gene containing both a GWAS hit and an MRE-SNP in this study is *N4BP1*. rs6500395, which is located in the first intron of *N4BP1*, has been associated with the response of rheumatoid arthritis patients to tocilizumab treatment [76], but this gene also contains an AGO-CLIP-supported C-type MRE-SNP proxy (rs1224) for miR-330-3p in its 3' UTR (Fig 4C).

In two cases, the absolute proxies of *cis*-eQTLs were located in the 3' UTR MRE of the nearby gene. rs3771570, which is associated with aggressive prostate cancer [74], is located in the intronic region of *FARP2* gene. However, it has a perfect proxy (rs1056801) within the 3' UTR of a gene next to it, *SEPT2*. The minor allele of rs1056801 disrupts the binding of cancer-associated miR-17-92 family members (Table 2, [46]), and aberrant expression of *SEPT2* has been reported in different tumor types [77]. As *SEPT2* is also the only gene influenced by a significant *cis*-eQTL effect in a corresponding LD block (FDR = 0.04, Z = 3.9 [21]), we propose that MRE-SNP-mediated alterations in the binding of miR-17-92 family miRNAs may be related to abnormal expression of *SEPT2* and could be a causative SNP in the GWAS locus identified by rs3771570.

In the esophageal squamous-cell carcinoma susceptibility region tagged by rs2239815 [78], we identified an MRE-SNP within the 3' UTR of *CCDC117*. This LD block contains two apparent candidate genes for cancer susceptibility (*XBP1* and *CHEK2*) (Fig 4D). Although all three of these genes are affected by *cis*-eQTLs, the largest effect of this LD block is associated with the *XBP1* gene (FDR < 0.01, Z = 23 [21]), casting doubt on the miRNA-mediated *cis*-eQTL mechanism.

In summary, we conducted a systematic and comprehensive survey to identify miRNA-mediated *cis*-eQTLs effects. Integrating data from different sources, we identified a number of potentially functional MRE-SNPs, serving as putative causative variants for allele-specific gene expression and for the development of complex traits.

## Supporting Information

S1 Dataset Unfiltered set of MRE-SNP associations. All reported *cis*-eQTL effects are significant after multiple testing correction (FDR < 0.05, [21]).
(ZIP)

S1 Fig Detailed analysis workflow. (a) *Cis*-eQTLs were acquired from BloodeQTLBrowser (FDR < 0.05). Perfect proxies (1000G CEU, R^2^ = 1) were added using the SNAP v2.2 webtool, and the resulting associations are referred to as SNP-probe associations. *Cis*-acting SNPs and their perfect proxies mapping into the 3’ UTR of corresponding *cis*-affected transcripts were used in subsequent analyses and are indicated as UTR-SNP-probe associations. (b) UTR-SNP-probe associations were intersected against resources containing information about *in silico* predicted MRE-SNPs (PolymiRTS uses Targetscan, miRSNP uses miRanda, and mrSNP uses a DIANA-based miRNA target prediction method). The resulting miR-SNP-probe associations (unfiltered set) were then filtered based on: i) overlap between all three of the target prediction methods, ii) their inclusion in a “blood-expressed” miRNA consensus list, and iii) their presence in the miRBase “high confidence” list. (c) Unfiltered and filtered sets of miR-SNP-probe associations were queried for concordance using the logic of miRNA-mediated regulation and classified as either concordant (C-type) and unconcordant (U-type).
(TIF)

S2 Fig The expression levels of 123 blood-expressed miRNAs detected from public datasets. Shown are expression levels of miRNAs detected by at least 10 reads in at least 6 blood samples out of 11. Points depict the median of RPM normalized by the number of reads mapped to the library, and error bars depict minimum and maximum RPM values using a log_10_ scale. Different colors indicate miRNAs detected in a corresponding number of samples, and the green dashed line corresponds to the overall median RPM.
(TIF)

S3 Fig Effect sizes in the different groups of miR-SNP-probe associations. The *cis*-eQTL effect sizes (log_10_[mean Z-scores] from the Westra *et al*. meta-analysis [21]) are shown for the following groups of MRE-SNPs: exclusively concordant (C-type), exclusively unconcordant (U-type), and ambiguous (having both C-type and U-type associations). (a) All MRE-SNPs, (b) only MRE-breaking SNPs, (c) only MRE-creating SNPs. The Wilcoxon-Mann-Whitney U-test was used to compare C-type and U-type groups.
(TIF)

S4 Fig Graph depicting a filtered set of miRNA-SNP-mRNA associations. The color of miRNA-SNP links shows the miRNA targeting probability, assessed using the TargetScan context+ score (grey- context+ score > 0, where the gradient from yellow to red shows the magnitude of a more negative context+ score change), and the line indicates the regulation concordance type: a continuous line is concordant (C-type), and a dashed line is unconcordant (U-type). The color of the SNP-gene links indicates the regulation concordance type: a red line is concordant (C-type), a blue line is unconcordant (U-type), and the intensity of the color indicates the *cis*-eQTL effect size (overall Z-score) from the Westra *et al*. meta-analysis [21], averaged over all detected SNPs in the LD block (R^2^ = 1). Multiple interactions between one SNP and one gene show either several Illumina probes detecting the same transcript or discordant effects of targeting miRNAs. Outlined SNPs correspond to those that are covered by reads in the AGO-CLIP-seq experiment. Outlined genes are validated targets of targeting miRNAs, as reported by miRTarBase, Tarbase, or miRecords databases.
(TIF)

S5 Fig Effect sizes for different groups of filtered miR-SNP-probe associations. Shown are *cis*-eQTL effect sizes (log_10_[mean Z-score] from the Westra *et al*. meta-analysis [21]) in different groups: C-type includes exclusively C-type MRE-SNPs, U-type includes exclusively U-type MRE-SNPs, and ambiguous-type includes MRE-SNPs having both C-type and U-type miRNA associations. (a) All MRE-SNPs grouped together, (b) only MRE-breaking MRE-SNPs, and (c) only MRE-creating MRE-SNPs. The Wilcoxon-Mann-Whitney U-test was used to compare C-type and U-type associations.
(TIF)

S6 Fig MRE efficiencies in the groups of filtered miR-SNP-probe associations. Shown are the mean measures of MRE efficiency for different groups: C-type (exclusively C-type MRE-SNPs); U-type (exclusively U-type MRE-SNPs); and ambiguous (MRE-SNPs having both C-type and U-type miRNA associations). (a) context+ score difference, (b) miRSVR score, (c) miRanda score, and (d) free binding energy. miRSVR scores were not available for every miRNA-target prediction, and, therefore, the ambiguous group is missing from the graph. The Wilcoxon-Mann-Whitney U-test was used to compare the groups of C-type and U-type associations.
(TIF)

S7 Fig Relationships between mean MRE efficiencies and *cis*-eQTL effect sizes. Mean MRE efficiencies are plotted: (a) mean context+ score difference, (b) miRSVR score, (c) miRanda score, and (d) free binding energy and effect sizes (log_10_[mean Z-score] from the Westra *et al*. meta-analysis [21]). Red dots represent exclusively C-type MRE-SNP-probe associations, blue dots represent exclusively U-type MRE-SNP-probe associations, and brown dots represent ambiguous MRE-SNP-probe associations. The Spearman correlation test was used to assess the correlation.
(TIF)

S8 Fig Pathways and Gene Ontology Biological Processes associated with the top 30 filtered miR-SNP-probes. Only pathways and processes in the top five predictions for at least one gene are shown on the heatmap. The color gradient from yellow to red indicates the Bonferroni-corrected p-value for function prediction. Gene Ontology Biological Processes, KEGG, and Reactome pathways with corresponding p-values were acquired from Gene Network (http://genenetwork.nl/genenetwork/, [42]).
(TIF)

S9 Fig Overlap between the top filtered miR-SNP-probe associations and *cis*-eQTLs from GTEx Consortium. The color of the cell depicts effect size and direction (beta-value), annotation bars on the left depict association concordance with the logic of miRNA-mediated regulation (C-type or U-type) and the effect direction in the study by Westra *et al*. Only the effect sizes for nominally significant tissue eQTLs are shown (uncorrected p < 0.05). Associations marked in bold contain SNPs in the validated miRNA binding sites. Top 30 filtered miR-SNP-probe associations and associations with validated miRNA binding sites are shown on this graph.
(TIF)

S1 Table Datasets used for defining the blood-expressed miRNA consensus list. Eleven samples from five separate studies were downloaded from Gene Expression Omnibus and analyzed with sRNABench v0.9.
(XLSX)

S2 Table AGO-CLIP-seq datasets downloaded from starBase v2.0 database (http://starbase.sysu.edu.cn/). (XLSX)

S3 Table Filtered set of MRE-SNP associations. Only associations where the MRE was predicted by all three target prediction algorithms and the targeting miRNA was present in both the blood-expressed consensus list of miRNAs and the miRBase “high confidence” list are reported in the filtered data set. ^a^ SNP effect on miRNA binding: D disrupts an MRE and C creates an MRE. ^b^ MAF is based on 1000G pilot 1 CEU population. ^c^ Number of *cis*-eQTL SNPs from the LD block (R^2^ = 1, 1000G pilot 1, CEU). ^d^ Z-scores from Westra *et al*. [21] are averaged for LD block (R^2^ = 1); FDR < 0.05 for all associations. ^e^ Association type: C-concordant; U-unconcordant.
(XLSX)

S4 Table Pathways and biological processes associated with the top 30 filtered SNP-miR-probe associations, sorted by context+ score. Gene Ontology Biological Processes, KEGG, and Reactome pathways with corresponding Bonferroni-corrected p-values (p < 0.05) were acquired from Gene Network (http://genenetwork.nl/genenetwork/, [42]). ^a^ SNP effect on miRNA binding: D disrupts an MRE and C creates an MRE. ^b^ MAF is based on 1000G pilot 1 CEU population. ^c^ Meta-analysis overall Z-scores from Westra *et al*. [21]. ^d^ Association type: C-concordant; U-unconcordant. ^e^ Validated: MRE presence in the TarBase, miRTarBase and miRecords.
(XLSX)

S5 Table Overlap of the filtered set of MRE-SNPs with the GWAS Catalog. (XLSX)
