# Supplementary figures and images for: Altered Gene Expression Associated with microRNA Binding Site Polymorphisms
Source: PLoS One. 2015 Oct 23;10(10):e0141351. doi: 10.1371/journal.pone.0141351 (PMC4619707; doi:10.1371/journal.pone.0141351)

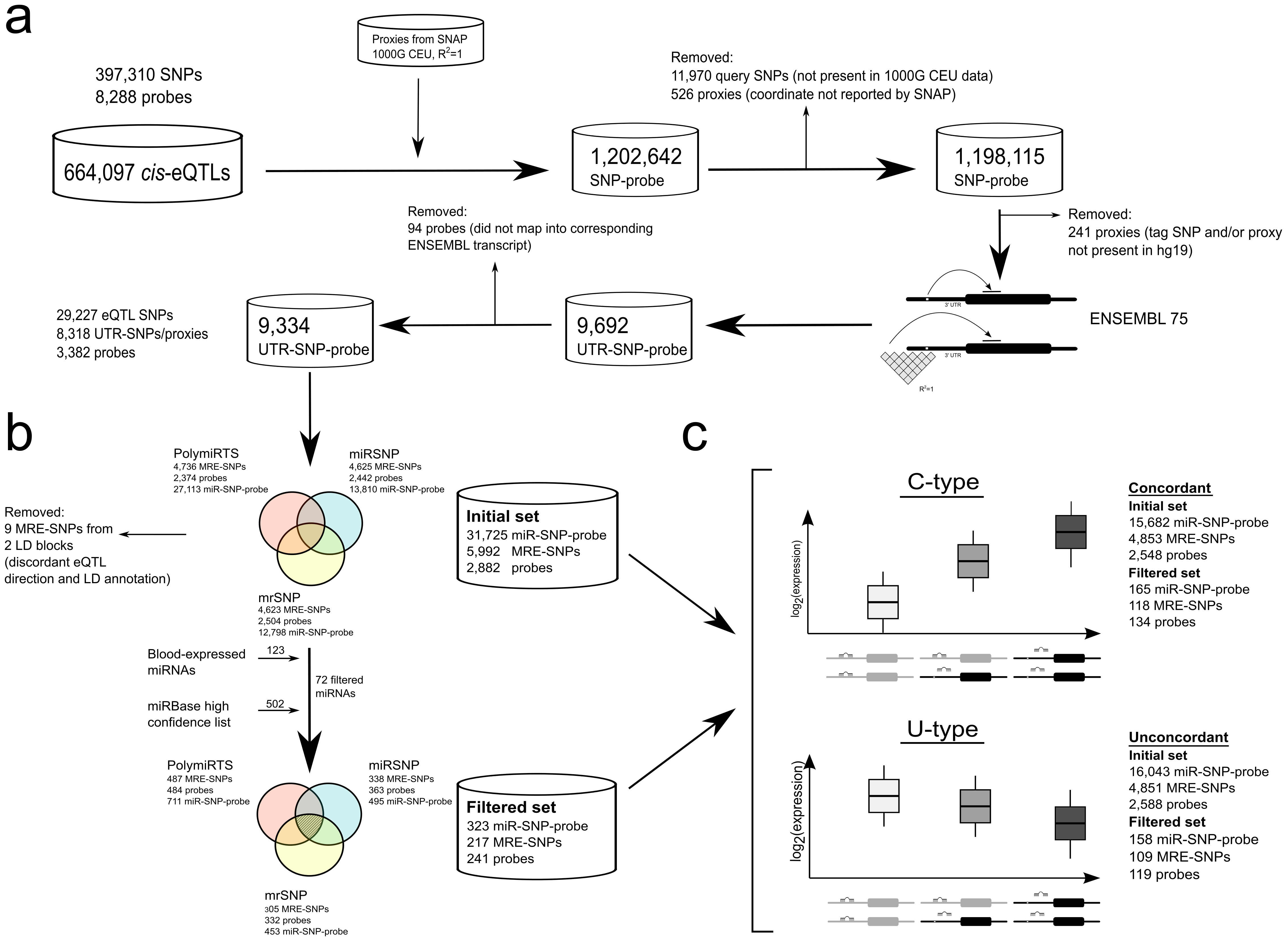

Supplement: S1 Fig — (a) Cis-eQTLs were acquired from BloodeQTLBrowser (FDR < 0.05). Perfect proxies (1000G CEU, R2 = 1) were added using the SNAP v2.2 webtool, and the resulting associations are referred to as SNP-probe associations. Cis-acting SNPs and their perfect proxies mapping into the 3’ UTR of corresponding cis-affected transcripts were used in subsequent analyses and are indicated as UTR-SNP-probe associations. (b) UTR-SNP-probe associations were intersected against resources containing information about in silico predicted MRE-SNPs (PolymiRTS uses Targetscan, miRSNP uses miRanda, and mrSNP uses a DIANA-based miRNA target prediction method). The resulting miR-SNP-probe associations (unfiltered set) were then filtered based on: i) overlap between all three of the target prediction methods, ii) their inclusion in a “blood-expressed” miRNA consensus list, and iii) their presence in the miRBase “high confidence” list. (c) Unfiltered and filtered sets of miR-SNP-probe associations were queried for concordance using the logic of miRNA-mediated regulation and classified as either concordant (C-type) and unconcordant (U-type). (TIF) [file pone.0141351.s002.tif]

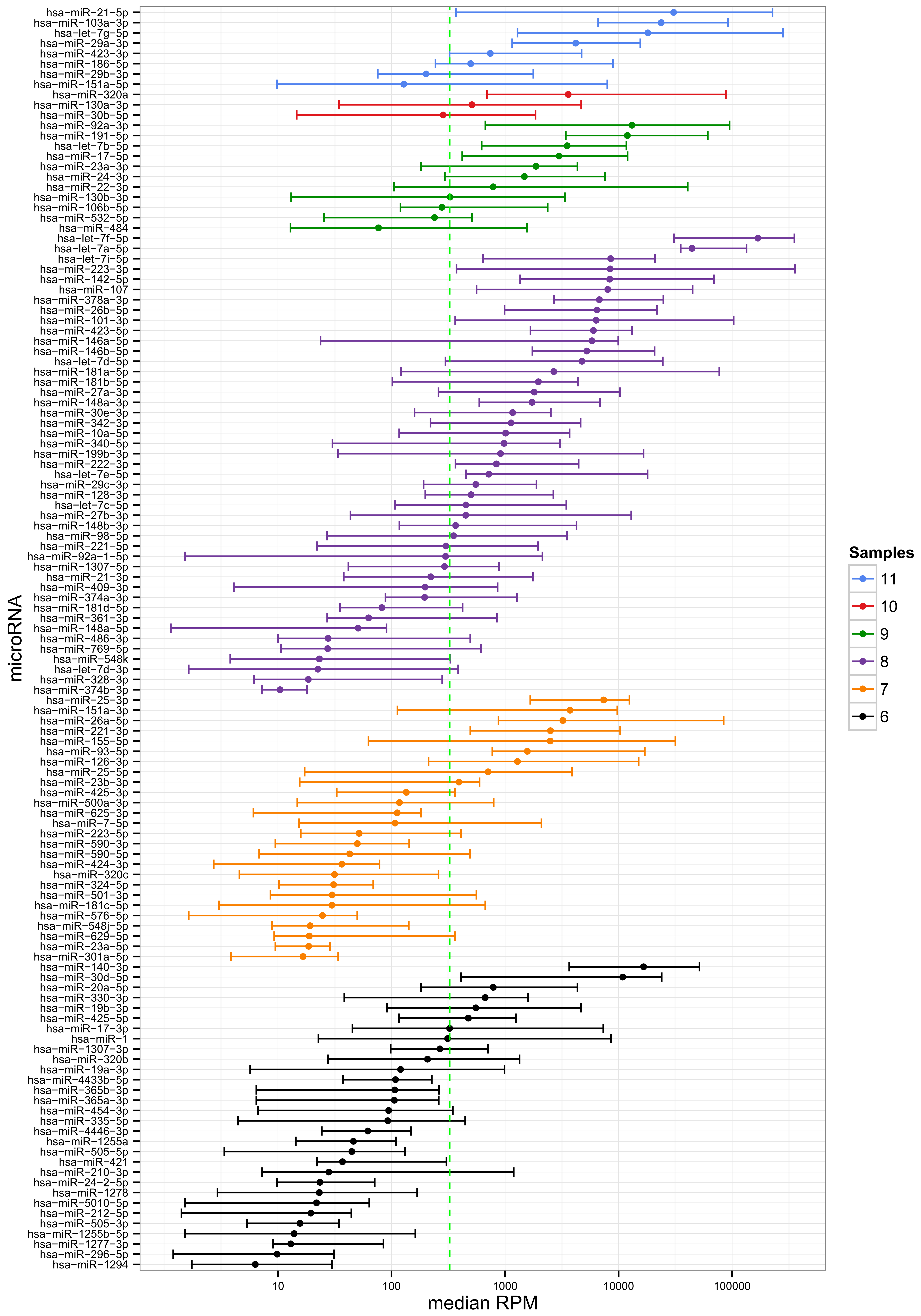

Supplement: S2 Fig — Shown are expression levels of miRNAs detected by at least 10 reads in at least 6 blood samples out of 11. Points depict the median of RPM normalized by the number of reads mapped to the library, and error bars depict minimum and maximum RPM values using a log10 scale. Different colors indicate miRNAs detected in a corresponding number of samples, and the green dashed line corresponds to the overall median RPM. (TIF) [file pone.0141351.s003.tif]

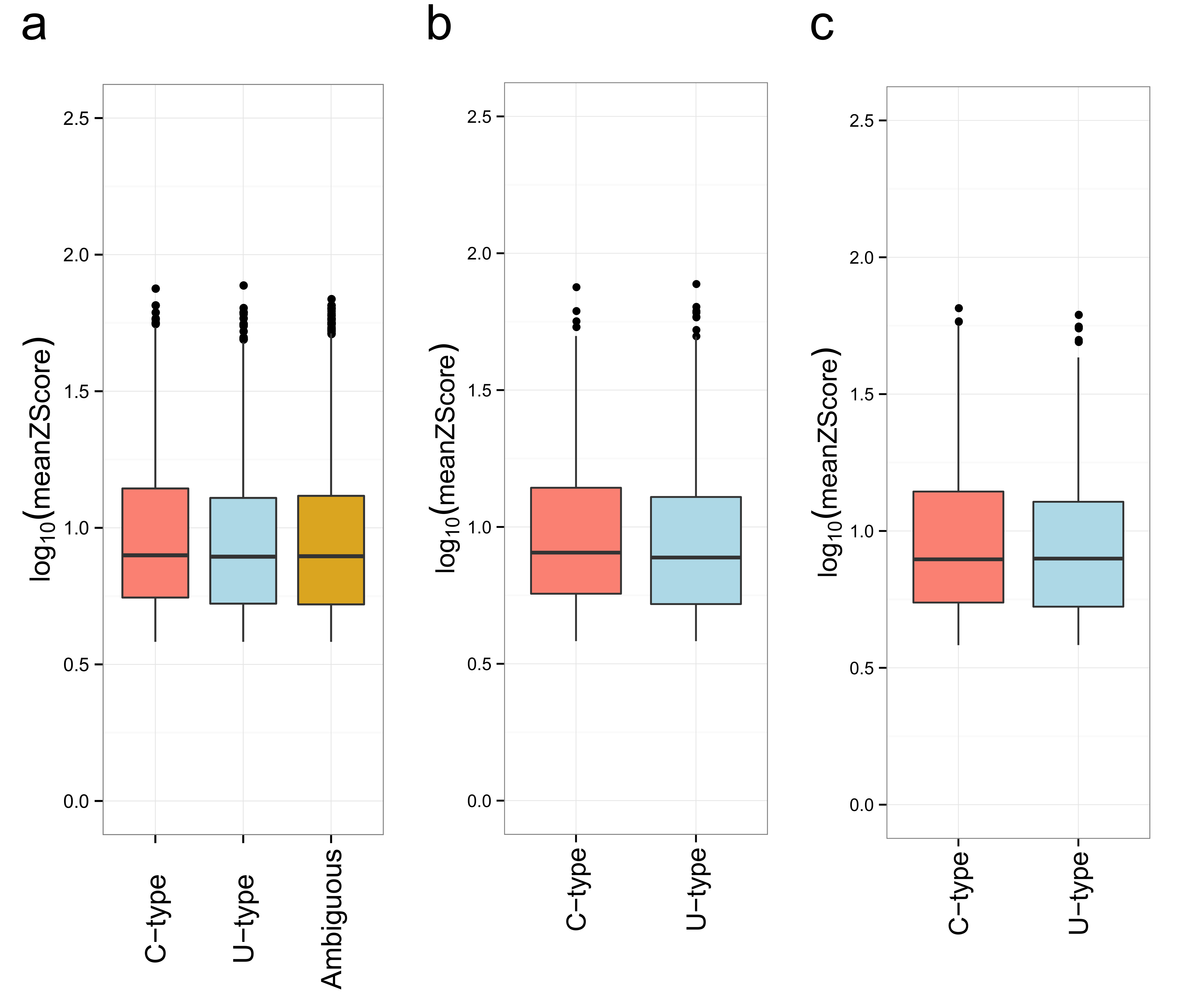

Supplement: S3 Fig — The cis-eQTL effect sizes (log10[mean Z-scores] from the Westra et al. meta-analysis [21]) are shown for the following groups of MRE-SNPs: exclusively concordant (C-type), exclusively unconcordant (U-type), and ambiguous (having both C-type and U-type associations). (a) All MRE-SNPs, (b) only MRE-breaking SNPs, (c) only MRE-creating SNPs. The Wilcoxon-Mann-Whitney U-test was used to compare C-type and U-type groups. (TIF) [file pone.0141351.s004.tif]

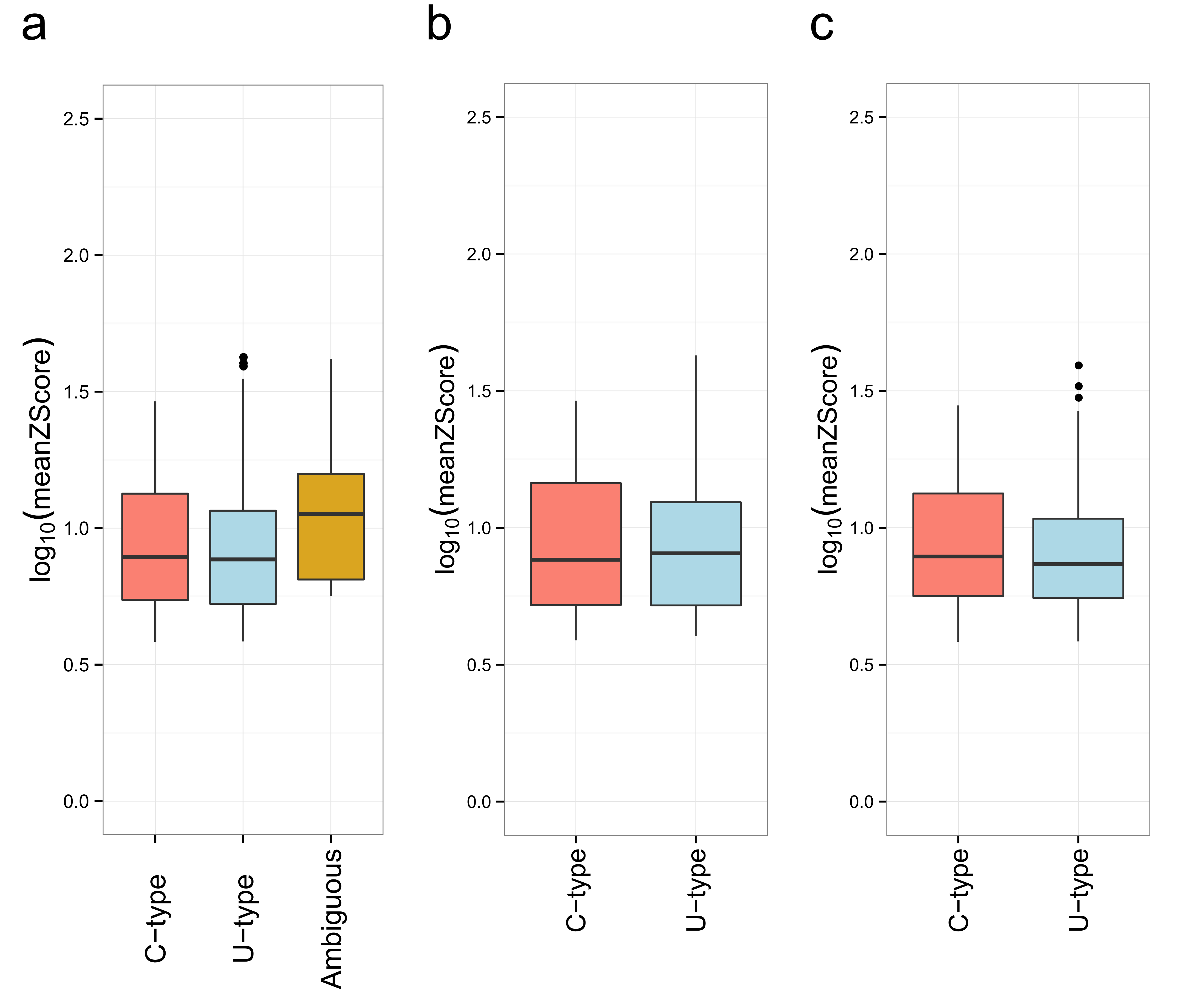

Supplement: S5 Fig — Shown are cis-eQTL effect sizes (log10[mean Z-score] from the Westra et al. meta-analysis [21]) in different groups: C-type includes exclusively C-type MRE-SNPs, U-type includes exclusively U-type MRE-SNPs, and ambiguous-type includes MRE-SNPs having both C-type and U-type miRNA associations. (a) All MRE-SNPs grouped together, (b) only MRE-breaking MRE-SNPs, and (c) only MRE-creating MRE-SNPs. The Wilcoxon-Mann-Whitney U-test was used to compare C-type and U-type associations. (TIF) [file pone.0141351.s006.tif]

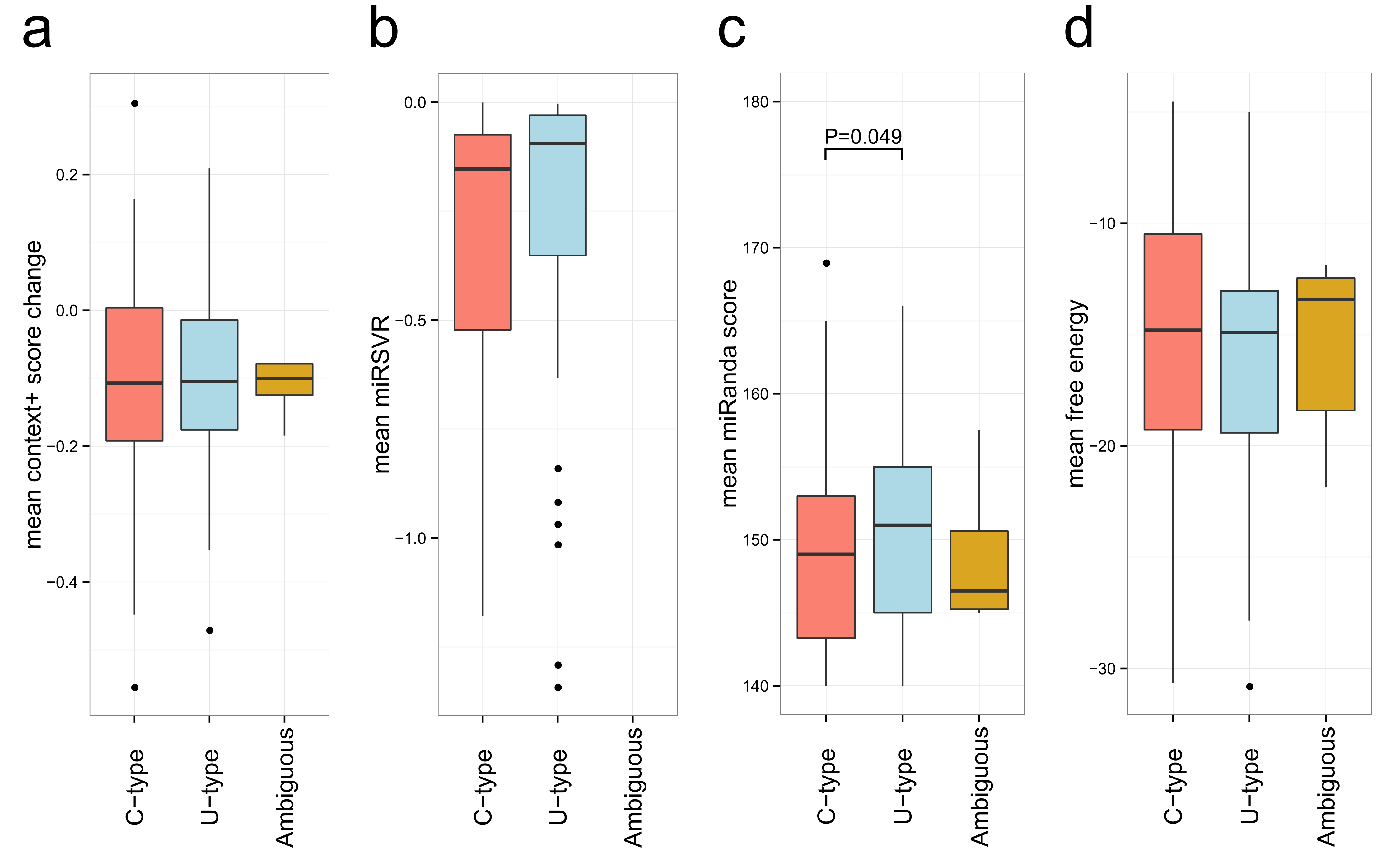

Supplement: S6 Fig — Shown are the mean measures of MRE efficiency for different groups: C-type (exclusively C-type MRE-SNPs); U-type (exclusively U-type MRE-SNPs); and ambiguous (MRE-SNPs having both C-type and U-type miRNA associations). (a) context+ score difference, (b) miRSVR score, (c) miRanda score, and (d) free binding energy. miRSVR scores were not available for every miRNA-target prediction, and, therefore, the ambiguous group is missing from the graph. The Wilcoxon-Mann-Whitney U-test was used to compare the groups of C-type and U-type associations. (TIF) [file pone.0141351.s007.tif]

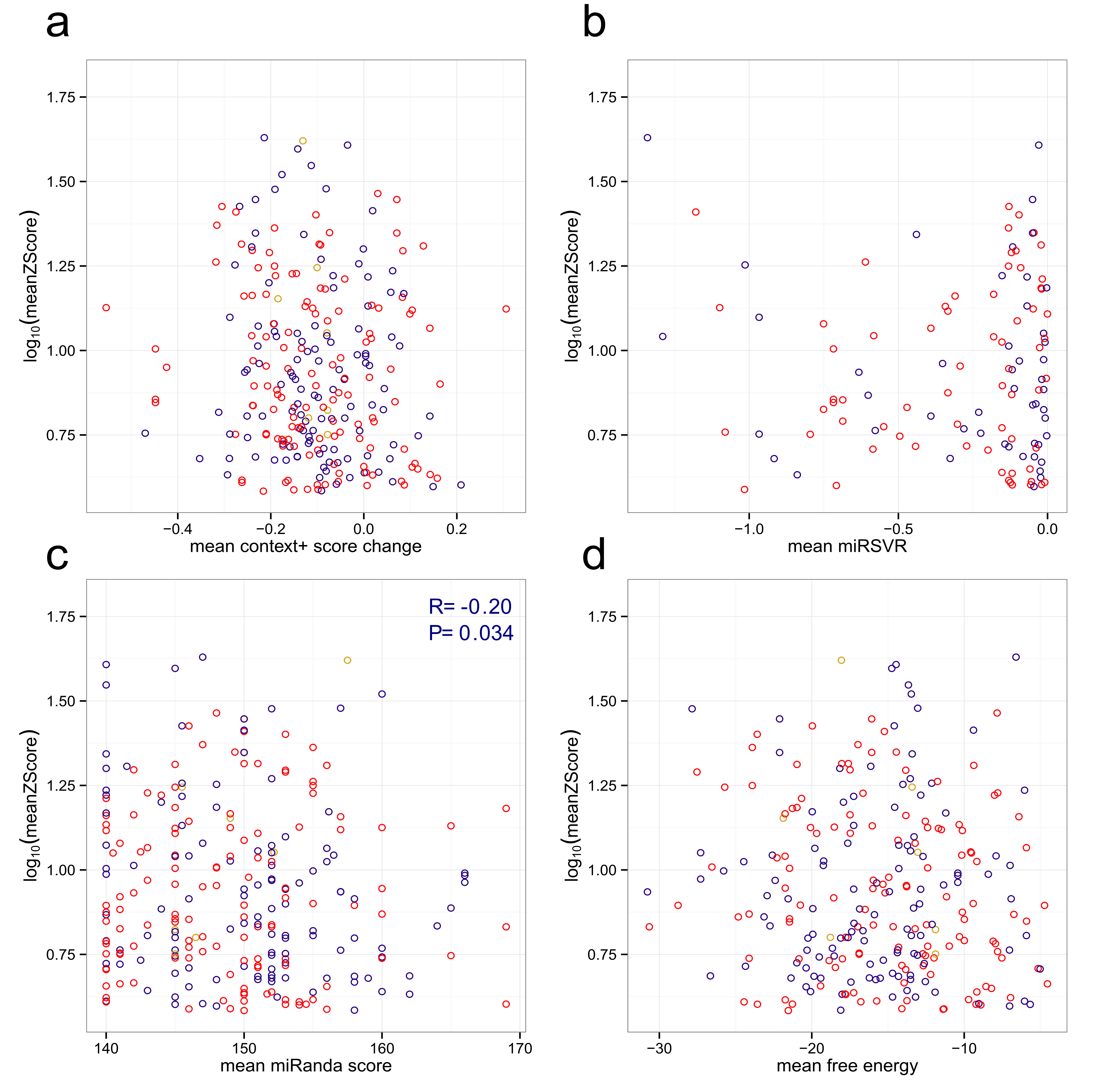

Supplement: S7 Fig — Mean MRE efficiencies are plotted: (a) mean context+ score difference, (b) miRSVR score, (c) miRanda score, and (d) free binding energy and effect sizes (log10[mean Z-score] from the Westra et al. meta-analysis [21]). Red dots represent exclusively C-type MRE-SNP-probe associations, blue dots represent exclusively U-type MRE-SNP-probe associations, and brown dots represent ambiguous MRE-SNP-probe associations. The Spearman correlation test was used to assess the correlation. (TIF) [file pone.0141351.s008.tif]

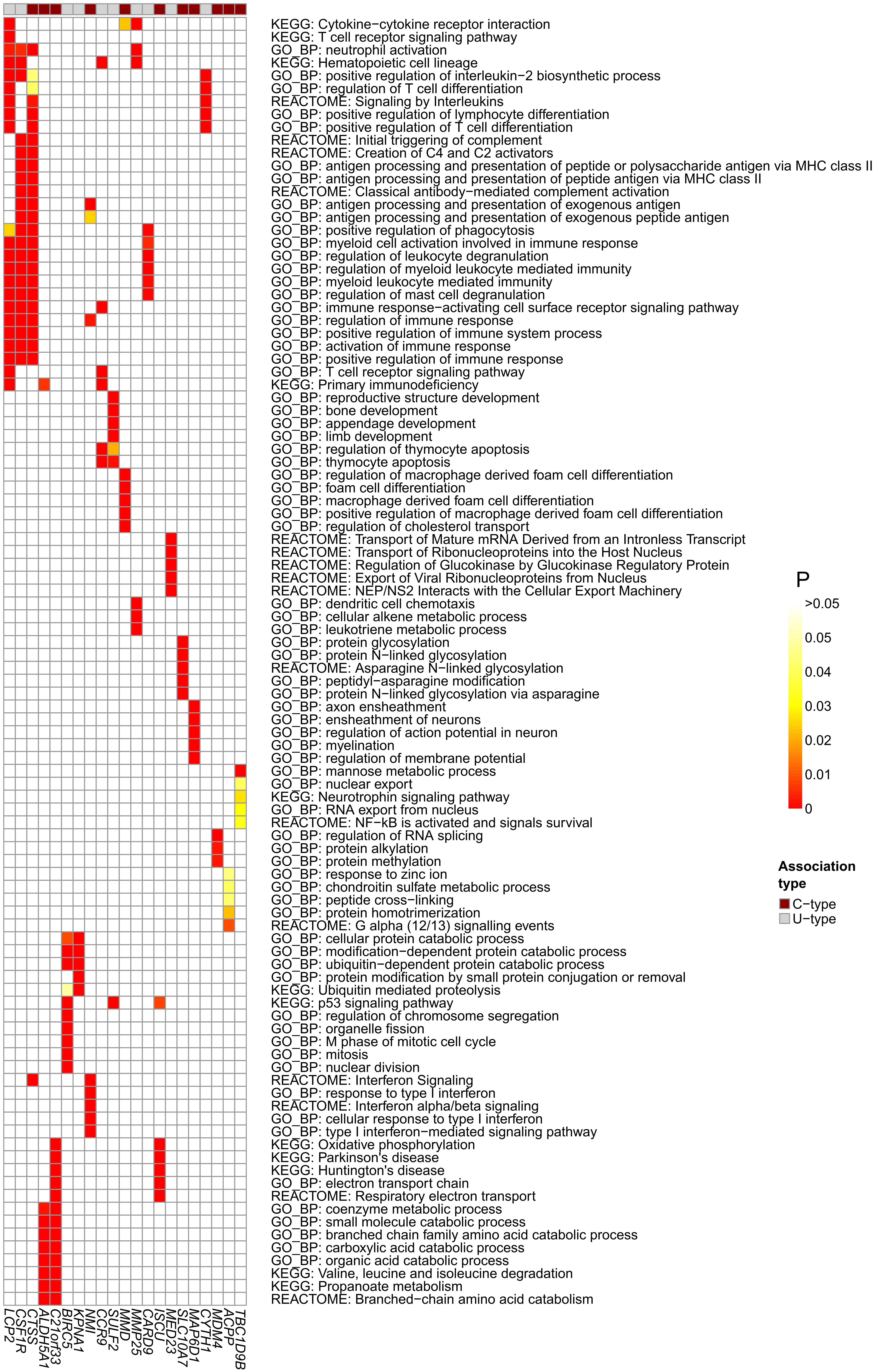

Supplement: S8 Fig — Only pathways and processes in the top five predictions for at least one gene are shown on the heatmap. The color gradient from yellow to red indicates the Bonferroni-corrected p-value for function prediction. Gene Ontology Biological Processes, KEGG, and Reactome pathways with corresponding p-values were acquired from Gene Network (http://genenetwork.nl/genenetwork/, [42]). (TIF) [file pone.0141351.s009.tif]

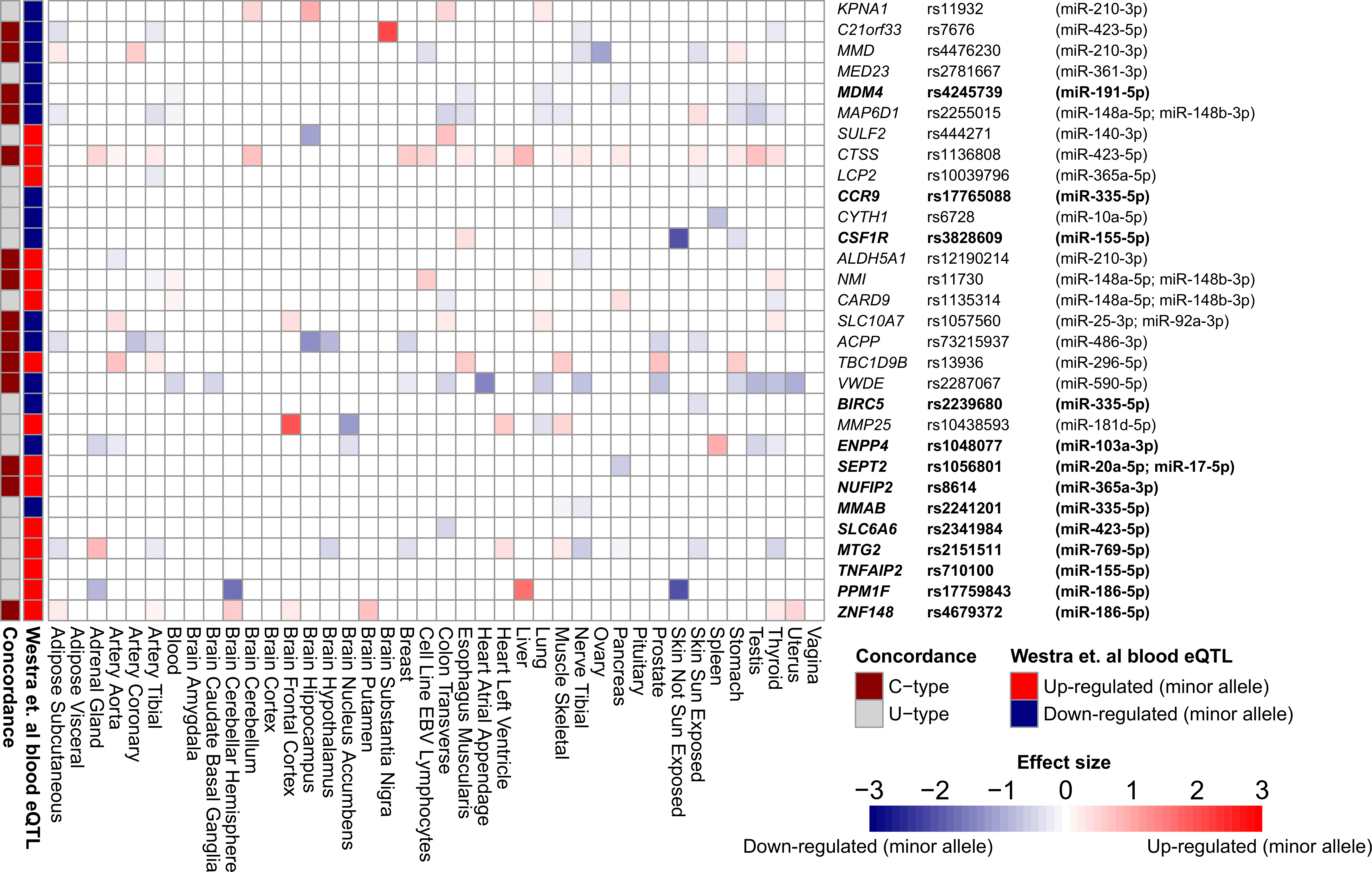

Supplement: S9 Fig — The color of the cell depicts effect size and direction (beta-value), annotation bars on the left depict association concordance with the logic of miRNA-mediated regulation (C-type or U-type) and the effect direction in the study by Westra et al. Only the effect sizes for nominally significant tissue eQTLs are shown (uncorrected p < 0.05). Associations marked in bold contain SNPs in the validated miRNA binding sites. Top 30 filtered miR-SNP-probe associations and associations with validated miRNA binding sites are shown on this graph. (TIF) [file pone.0141351.s010.tif]
